# Interwoven oppression: A qualitative study of the contribution of racism and sexism to co‐occurring PTSD and substance use disorder

**DOI:** 10.1002/ajcp.70104

**Published:** 2026-09-08

**Authors:** Mallet R. Reid, NiCole T. Buchanan, Thalia S. Epps, Edgar Jaimes‐Bautista, Jaidyn J. Choi, Enrique Romero, Ximena Murillo

**Affiliations:** ^1^ Department of Family Medicine Michigan State University East Lansing Michigan USA; ^2^ Department of Psychology Michigan State University East Lansing Michigan USA; ^3^ Michigan State University East Lansing Michigan USA; ^4^ United 4 Change Center Houston Texas USA

**Keywords:** addiction, health equity, multiple jeopardy, posttraumatic stress disorder, racism, sexism, substance use disorder, trauma

## Abstract

Racism and sexism are associated with posttraumatic stress disorder (PTSD) and substance use disorder (SUD), yet few studies examine their role in co‐occurring PTSD/SUD. Through qualitative interviews with 23 people of color with PTSD/SUD, we found that racism and sexism influence the onset of men and women of color's PTSD/SUD in gendered ways. Women reported that sexism played a role in their exposure to sexual violence, and that concerns around being blamed or dismissed when reporting sexual trauma because of their gender and race, they used substances to cope. Men reported that racism rendered them vulnerable to gang, gun, and interpersonal violence. Sexism via gender stereotypes made them feel like they had to “tough it out,” and the racist criminalization meant that they could not seek help for their traumas or substance use. Thus, each group's PTSD/SUD onset occurred through racism and sexism, yet in distinctly gendered ways. Our findings align with multiple jeopardy theory, which suggests that interlocking oppressions render people vulnerable to harm in unique ways. They highlight the need for community psychologists to center the experiences of people of color and to study, prevent, and treat the unique challenges giving rise to their PTSD/SUD.

## Highlights

- Racism and sexism contribute to the onset of posttraumatic stress disorder (PTSD)/substance use disorder (SUD) in people of color.
- For women of color, gender‐based violence, invalidation, and victim blaming led to their PTSD/SUD.
- For men of color, interpersonal trauma, mental health stigma, and criminalization led to PTSD/SUD.
- Currently, people of color and their challenges are neglected by PTSD/SUD research.
- Our findings reveal the need to address people of color's challenges linked to PTSD/SUD.

## INTRODUCTION

In the United States (US), resources, opportunities, and power are inequitably distributed across groups in ways that privilege White men and disadvantage men and women of color (Homan, 2019; Ray, 2023). These systemic inequities, often conceptualized as structural racism and structural sexism, are reinforced through policies, laws, and social norms and contribute to persistent, measurable health disparities among people of color (Bailey et al., 2017; National Academies of Sciences, Engineering, and Medicine, 2024; Yearby, 2018). These inequities contribute to health disparities through interconnected structural and interpersonal processes. In this study, we use the term structural racism/sexism to refer to systemic inequities embedded within institutions, policies, laws, and social arrangements that differentially distribute risk, resources, power, and vulnerability across groups. In contrast, interpersonal racism/sexism refers to person‐to‐person manifestations of oppression, including discrimination, microaggressions, victim blaming, stereotyping, and testimonial dismissal (Brown et al., 2025; Collins & Bilge, 2020). Although analytically distinct, these forms of oppression are mutually reinforcing, as structural inequities often shape the interpersonal conditions people encounter in daily life.

To illustrate, due to the legacies of Jim Crow and Redlining, neighborhoods in the United States remain segregated by race (Logan & Parman, 2025). Those neighborhoods predominantly comprised of people of color endure concentrated economic disinvestment and over‐ and under‐policing, lowering residents' access to beneficial social resources while heightening their exposure to various forms of community and state violence (Bradford et al., 2024; Sewell & Jefferson, 2016). These issues have been implicated in people of color's heightened rates of trauma exposure (Bradford et al., 2024). Further, there is a continuous decline in healthcare settings and resources in communities of color (King et al., 2022; Ko et al., 2014; Tung et al., 2024). In contrast, there is a growing density of alcohol and cannabis retail outlets in communities of color (Berke et al., 2010; Firth et al., 2022). Thus, people of color's access to clinical services is constrained, and they are funneled toward substance use as a potential coping mechanism for the traumas that they are exposed to (Skewes & Blume, 2019).

At the interpersonal level, national data show that up to 75% of Black, Latiné, and Asian people report experiencing some form of racial discrimination in their lifetime (Lee et al., 2019). These include experiences with overt racism and microaggressions (subtle yet harmful identity‐related slights; Sue & Spanierman, 2020). Similarly, women report frequent encounters with sexism (estimated around two a week; Swim et al., 2001), which include sexual objectification and gender role stereotyping (Basford et al., 2014). As with the systemic issues mentioned above, these interpersonal issues have also been linked to trauma and adverse substance use for oppressed group members (Farahmand et al., 2020; Stockman et al., 2015; Williams et al., 2021).

Notably, Black Feminist scholars and activists have argued for decades that racism and sexism converge in ways such that, although they affect all people of color, their manifestations and impacts vary by gender (Collins & Bilge, 2020; Crenshaw, 1989; King, 1988). For instance, the over‐ and under‐policing of communities of color noted above has uniquely and significantly increased Black, Latiné, and Indigenous men's risk of arrest for drug possession (Alexander, 2010), despite evidence that all racial groups use drugs at similar rates (National Survey on Drug Use and Health [NSDUH], 2020). Additionally, this issue has reduced men of color's security against exposure to gang and gun violence (Alexander, 2010). In contrast, due to over‐ and under‐policing, Black, Latiné, and Indigenous women are more likely to endure gender‐based violence from both police and civilians and face systemic barriers to justice when victimization occurs (Ritchie & Jones‐Brown, 2017). Thus, while people of color face a heightened risk for trauma due to structural and interpersonal racism and sexism, they endure these issues differently by gender.

### Racism and sexism's contributions to posttraumatic stress disorder and addiction

Given the above, it is perhaps unsurprising that investigations into the health consequences of racism and sexism in relation to posttraumatic stress disorder (PTSD) and substance use disorder (SUD) are increasing, albeit mainly at the individual level. Researchers have found that approximately 70% of reported associations between racial discrimination and trauma symptoms were significant and positive, with correlations often in the moderate‐to‐strong range (*r* = .45–.68). Further, longitudinal evidence from Sibrava et al. (2019) indicates that, among those with PTSD, higher levels of reported discrimination were associated with lower rates of symptom remission and a greater likelihood of a chronic disorder trajectory. Moreover, neuroimaging research shows that racial discrimination is associated with altered neural functioning in threat‐related circuits among Black women with PTSD (Fani et al., 2021), suggesting that exposure to discrimination may exacerbate neurobiological vulnerability among people with PTSD, which could partially explain people of color's heightened PTSD symptom severity and chronicity. Similarly, sexism‐related violence, including sexual, domestic, and intimate partner violence, substantially contributes to PTSD (Fedina et al., 2022; Rodríguez et al., 2009). Further, women often exhibit more acute PTSD symptoms than men (Olff, 2017), plausibly due to the nature of the trauma they experience (Kessler et al., 2014) and the cumulative burden of gendered expectations, discrimination, and caregiving demands they endure (Homan, 2019).

Additionally, racial discrimination has been widely studied as a risk factor for SUD among people of color (Downey & Chang, 2024). Meta‐analytic evidence indicates a consistent, small‐to‐moderate association between discrimination and SUD (*r* = .25; 95% confidence interval [CI] = 0.14–0.36), though the magnitude of effects varies across studies and populations (Gates et al., 2025). Most studies using large‐scale and national survey data have consistently shown an association between exposure to discrimination and SUD, often in a dose‐response fashion (Chae et al., 2008; Cheadle & Whitbeck, 2011; Glass et al., 2020; Hunte & Barry, 2012; Whitbeck et al., 2004). Meta‐analytic evidence also links discrimination to hazardous substance use, which refers to patterns of use that meet a clinical threshold for potential harm (Gates et al., 2025). Therefore, given that exposure to discrimination is associated with greater substance use and more harmful patterns of use, discrimination may increase people of color's risk of developing SUD. Finally, although less research has explicitly linked gender discrimination and SUD, sexism‐related experiences also heighten women's risk of SUD, as substance use increases in proportion to their exposure to gender‐based violence (Cohen et al., 2013).

### Knowledge gaps and rationale for the current study

Despite growing awareness of oppression's role in PTSD and SUD, no studies have examined how racism and sexism influence the onset of co‐occurring PTSD and SUD (PTSD/SUD). Moreover, no studies have drawn from a Black feminist tradition to explore whether and how converging racism and sexism may affect co‐occurring PTSD/SUD differentially by gender. Yet, as we detailed above in relation to the differential effects of over‐ and under‐policing on men and women of color, racism and sexism could interact in ways that may create distinct pathways to PTSD/SUD among men and women of color.

These oversights are concerning because it has been argued that men and women of color may have a disproportionately high burden of PTSD/SUD (Reid et al., 2025), and they endure more severe and chronic PTSD/SUD (Brown et al., 2022; Ruglass & Yali, 2019; Ruglass et al., 2016; Schäfer et al., 2019). Further, given the established impact of racism and sexism on PTSD and SUD, it is plausible that these issues may also have an impact on PTSD/SUD. Investigating the potential link between race and gender oppression and PTSD/SUD is essential for refining research and clinical training to address health inequities.

### Theoretical framework

This study was guided by intersectionality and feminist standpoint epistemology (FSE), both of which informed how we conceptualized the relationship between oppression and PTSD/SUD among people of color. Intersectionality, rooted in Black feminist scholarship, emphasizes that systems of oppression such as racism and sexism do not operate independently but interact to shape social experiences and vulnerabilities in distinct ways across social positions (Collins & Bilge, 2020; Crenshaw, 1989). This framework informed our attention to how race and gender may jointly shape trauma exposure, coping processes, and engagement with care among men and women of color.

Feminist standpoint epistemology emphasizes that marginalized groups possess critical forms of situated knowledge derived from their social positions and lived experiences (Sprague, 2016). Accordingly, this study centered participants' interpretations of how racism, sexism, trauma, and substance use intersected in their lives. These frameworks shaped the study's interview approach, analytic focus, and interpretation of findings by orienting attention toward structural oppression, lived experience, and gender‐differentiated pathways into PTSD/SUD.

## METHOD

Our university's Institutional Review Board (IRB) approved this study, and all participants provided verbal informed consent to participate (IRB STUDY00009375).

### Recruitment

We recruited participants from community mental health clinics in Michigan, New Mexico, Georgia, and California. MRR had an established community‐engaged partnership with each clinic, stemming from prior program evaluation work. These settings were also chosen because they enabled wide recruitment from various parts of the United States. These clinics serve anywhere from 100 to several thousand people every year. We created a recruitment flyer, and each clinic displayed it from November 2023 through March 2024. Participants demonstrated interest by responding to the flyer's QR code, phone number, or email. Participants were eligible to participate if: (a) they identify as a person of color; (b) they have been diagnosed with co‐occurring PTSD and SUD by a clinician; (c) they have received clinical treatment for PTSD, SUD, or PTSD/SUD; (d) they spoke English; and (e) they were ≥ 18 years old.

### Participants

Thirty‐one people responded to the recruitment flyer and completed a brief questionnaire to determine eligibility. Eight people were excluded because they did not have a PTSD/SUD diagnosis. The remaining 23 were diagnosed with PTSD/SUD by a clinician and had or were currently receiving therapy for PTSD, SUD, or PTSD/SUD. Eight received SUD treatment only, four received PTSD treatment only, and eleven received PTSD/SUD treatment.

Participants were from Michigan (*n* = 4), Georgia (*n* = 7), New Mexico (*n* = 4), and California (*n* = 8; Table 1). This sample included 16 participants who identified as women, including two transgender women. Ten participants identified as Latine, seven identified as cisgender Latinas, and one identified as a transgender Latina; eight were Black, including four women; four were multiracial; and one was Asian. Among multiracial participants, two stated that they are racialized Black, one shared that she is racialized as White, and one as Latina. Their ages ranged from 18 to 52 (m = 28, SD = 8.24). Three participants had a master's degree, eight had a college degree, and 12 had a high school education. Twenty participants identified as heterosexual, one as lesbian, one as queer, and one as bisexual.

**Table 1:** Participant demographics.

Participant*	Location	Race/Ethnicity	Gender	Sexuality	Age
Audre	Michigan	Black & Latine (Racialized Black)	Female	Queer	23
Malcom	Georgia	Black	Male	Heterosexual	29
Martin	Georgia	Black	Male	Heterosexual	37
Angela	Georgia	Black	Female	Heterosexual	36
Nelson	Georgia	Black	Male	Heterosexual	33
Rosa	Georgia	Black	Female	Heterosexual	28
Fannie	Georgia	Black	Female	Heterosexual	30
Bell	Georgia	Black & White (Racialized Black)	Female	Heterosexual	33
Geena	California	Asian	Transgender Female	Lesbian	19
Bamby	California	Latine	Transgender Female	Heterosexual	29
Dolores	California	Latine	Female	Heterosexual	28
Cesar	California	Latine	Male	Heterosexual	19
Sylvia	California	Latine	Female	Heterosexual	26
Eunice	California	Latine & White (Racialized White)	Female	Heterosexual	38
Gloria	California	Latine	Female	Queer	39
Elvia	California	Latine	Female	Heterosexual	41
Oscar	New Mexico	Latine	Male	Heterosexual	26
Alicia	New Mexico	Black	Female	Heterosexual	24
Maritza	New Mexico	Latine	Female	Heterosexual	42
Isabel	New Mexico	Latine & Indigenous (racialized Latine)	Female	Heterosexual	50
DeRay	Michigan	Black	Male	Heterosexual	22
Ignacio	Michigan	Latine	Male	Heterosexual	32
Leslie	Michigan	Latine	Female	Heterosexual	37

* All participant names are pseudonyms.

### Procedure

Twenty participants were interviewed via HIPAA‐compliant Zoom, and three in person. Of note, each person who qualified for the study and who lived in the same area as the research team was invited to participate in either an in‐person interview or a Zoom interview. Most chose to join over Zoom, citing convenience as their reason. The three in‐person participants chose to meet in person, citing a preference for in‐person conversation over Zoom. We found no material differences in demographics or experience between those who elected to participate in person and those who elected to participate over Zoom.

Each interview began with rapport building, a discussion of confidentiality, and a brief conversation about the interviewer's positionality. Interviews focused on participants' recovery journeys and whether or how race and gender shaped their experiences with PTSD, substance use, and clinical care. Probing questions were specific to each interview. Interviews averaged about 1 h, ranging from 40 to 90 min, and included a 5‐min break. With permission, interviews were recorded, transcribed, and sent to participants for verification. Each participant received $75. Before the interviews, since TSE was an undergraduate with limited interview experience, MRR held four 1‐h training sessions, including mock interviews and skill‐building exercises. Afterward, MRR and TSE conducted interviews, transcribed the data, and coded it. Participants reviewed and confirmed the accuracy of their transcripts. EJ‐B assisted with transcription review and participated in discussions regarding emerging themes, analytic interpretation, and study procedures throughout the project.

After completing the first round of interviews (*n* = 23), MRR and TSE collaboratively developed a follow‐up interview guide based on emergent themes and analytic memos from initial coding. The follow‐up interviews were designed to deepen our understanding of themes, clarify ambiguous narratives, and ensure that our developing interpretations aligned with participants' lived experiences, which is a process consistent with feminist and reflexive qualitative research traditions. Particular attention was given to patterns or ambiguities related to how participants navigated race, gender, trauma, and substance use, especially where more elaboration or clarification could strengthen analytic interpretation. For example, whereas some participants discussed using substances to cope with trauma and specified how identity shaped that experience in their first interview, others did not. Therefore, follow‐up questions included: “Do you think your race or gender had anything to do with how you coped?”. When participants who already identified that race or gender were relevant to their experiences, we asked them to elaborate. These responsive questions enabled deeper exploration of the intersectional dynamics at play in participants' trauma, coping strategies, and engagement with care.

Using a random number generator, five participants were selected from the original sample to complete a second interview. This purposive‐random strategy allowed for conceptually rich follow‐up while avoiding selection bias. The follow‐up interviews, which lasted approximately 30–45 min and were all completed over Zoom, employed a semi‐structured format tailored to each participant's content from their initial interview. This approach enabled the researchers to confirm emerging themes, explore unresolved questions, and ensure analytic saturation. Participants again reviewed their transcripts for accuracy and received $75 compensation. A total of 28 interviews were conducted.

### Analysis

MRR and TSE conducted open‐ended, semi‐structured qualitative interviews. Interviews were designed and guided by a semi‐structured protocol developed through an iterative, reflexive process grounded in feminist and critical paradigms. We began by identifying core domains based on gaps in the literature, particularly the limited attention to how race, gender, and structural oppression shape PTSD/SUD trajectories. The guide was then refined through discussions between the authors, who drew on both the existing literature and their experiences working with marginalized communities. The final protocol centered on participants' recovery journeys, including questions about trauma exposure, substance use onset, experiences with mental health and substance use treatment, and whether or how they believed race, gender, or other identities shaped these experiences. All interviews were conducted responsively such that while the guide ensured core themes were addressed, probing questions were adapted in real time based on what participants shared, consistent with a feminist commitment to relational interviewing and participant‐led meaning‐making (Miles et al., 2020; Sprague, 2016).

Whereas conventional interviewing techniques emphasize interviewer control and detachment (Sprague, 2016), this study was framed by feminism, which emphasizes reciprocity and relational engagement between researcher and participant (Sprague, 2016). To reflect this orientation, we used a responsive, semi‐structured interviewing model developed by Miles et al., 2020, which allowed us to adapt questions in real time based on participants' responses. Our thematic analysis was grounded in feminist standpoint epistemology and intersectionality, which prioritize participants' lived experiences as situated, partial, and legitimate knowledge, particularly as shaped by race, gender, class, and trauma histories (Sprague, 2016). This orientation influenced how we approached participants and how we interpreted the data, centering what participants emphasized as meaningful while attending to how power, oppression, and identity shaped those meanings.

### Positionality and reflexivity

We situate researcher positionality as one component of reflexive practice and link reflexive reflection to strategies used to enhance trustworthiness across data collection, analysis, and interpretation.

#### Researcher positionality

Feminist researchers position themselves in their studies to reveal how their social locations and experiences influence their research, promoting transparency and accountability in data interpretation (Sprague, 2016). Each author is a person of color with a feminist orientation and varying research and clinical education levels. The primary investigators for this study (MRR and NTB) are community psychologists and licensed clinicians with overlapping areas of expertise. MRR specializes in feminist standpoint theory, qualitative research methods, and substance use. NTB specializes in intersectionality theory, social justice research, and trauma. Combined, their foci provided this study's theoretical and methodological grounding. TSE, EJ‐B, and JJC were undergraduate students, each with academic training and a strong personal commitment to social justice and health equity. ER was a medical student who had received certificate training for working with underserved and marginalized populations. ER and JJC joined the project after primary data collection and coding were complete. Although they were not involved in interviewing or formal coding, they reviewed analytic memos, emergent themes, and manuscript drafts and provided critical feedback regarding interpretation, clarity, and implications during the writing process.

#### Reflexive practices across the study

MRR and TSE maintained a reflexive journal to document thoughts, feelings, and assumptions about the data, shared these weekly, and met weekly to discuss or adjust the developing findings (Miles et al., 2020). Additionally, MRR and TSE sought disconfirming evidence through follow‐up and feedback interviews and peer debriefing to counter confirmation bias and enrich the framework. They maintained an audit trail to track the application of rigor criteria and provided a de‐identified audit trail to a panel of auditors (Miles et al., 2020). Moreover, MRR debriefed NTB weekly regarding interview dynamics, emerging interpretations, coding decisions, assumptions, and potential blind spots in the analytic process. Although MRR and TSE conducted the primary coding and thematic development, broader team discussions with NTB and EJ‐B were used to critically examine interpretations, identify potential overreach, and consider alternative explanations grounded in participants' narratives. Finally, XM assisted us with figure development. Her work allowed us to visualize our findings.

In addition to these procedural reflexive practices, reflexivity shaped analytic interpretation. Rather than “bracketing out” our assumptions, we engaged in reflexive memoing and collaborative discussions to explore how our social positions shaped the meaning we made of participants' narratives. In one instance, early in the analysis, we coded several women's stories under the umbrella of “hesitation to seek help.” However, upon reflection, we recognized that this framing subtly implied individual‐level hesitation rather than capturing the structural forces, like testimony dismissal and victim blaming, that participants described. A feminist lens helped us reframe the theme to center the role of systemic silencing and powerlessness, rather than misattributing the issue to an individual‐level issue. In this way, feminist reflexivity allowed us to interrogate how dominant assumptions could distort meaning and reorient our coding toward the structural and intersectional forces participants were naming. Furthermore, when coding, we sought patterns across individuals and social positions, inquiring how themes might have varied by gender, race, or other axes of identity.

### Trustworthiness

In qualitative research, trustworthiness concerns the credibility and integrity of descriptions, conclusions, and interpretations, not whether other researchers would have reached the same conclusions (Miles et al., 2020). The primary threat to the trustworthiness of this study lies in the differences in researcher and participant positionalities. Therefore, MRR and TSE listened to each other's interviews throughout the interview process and encouraged critique of each other's interpretations of the data based on researcher positionality. Moreover, we maintained an audit trail, sought contrary evidence throughout the project, and reviewed the study findings with the entire research team.

Next, we addressed common threats to trustworthiness, such as poorly detailed data or “thin description” (Miles et al., 2020). We conducted semi‐structured, open‐ended interviews and reinterviewed participants to gather their perspectives on emerging categories aimed at combating thin descriptions. Additionally, we created detailed transcriptions and memos and linked the raw data to themes using a data display matrix.

### Analysis

We conducted our analysis using the systematic framework outlined by Miles et al. (2020), which emphasizes iterative cycles of data condensation, data display, and conclusion drawing/verification. Our analytic process involved multiple rounds of descriptive and pattern coding, analytic memoing, and iterative team‐based interpretation. We used Dedoose to manage the qualitative data and applied descriptive coding to summarize salient segments of text using concise labels (Saldana, 2021). MRR and TSE collaboratively refined the codebook through iterative discussion and consensus‐building, reducing an initial set of 32 codes and 15 subcodes to a final set of 12 codes. Intercoder agreement was approximately 83%.

Following descriptive coding, we engaged in pattern coding and analytic categorization to identify higher‐order explanatory patterns across participants' narratives. Rather than treating themes as topic summaries, we developed analytic categories that captured how race‐ and gender‐based oppression structured participants' exposure to trauma and led to their PTSD, their coping responses, and the conditions under which substance use escalated into SUD. These categories were revised to eliminate overlap, clarify conceptual boundaries, and maintain alignment with the study's focus on race, gender, trauma, and substance use.

Through this iterative process, we identified a higher‐order analytic theme, multiple jeopardy as a structuring condition of PTSD/SUD onset, and a set of gender‐differentiated subthemes that specify the mechanisms through which racism and sexism operated in participants' lives. Participant‐engaged meaning‐making informed the development and validation of these subthemes, ensuring that our analytic interpretations remained grounded in their accounts.

## RESULTS

Twenty‐one participants identified a link between their race, gender, and PTSD/SUD. Below, we demonstrate how racism and sexism interact with these factors to impact participants' PTSD/SUD (Table 2). We use participant quotations to illustrate the analytic themes. Pseudonyms are used throughout, and italics reflect participants' original emphasis. Brackets within quotations were added by the researchers to clarify contextual meaning when necessary. Across genders, violence, silence, and coping were recurring patterns; however, multiple jeopardy shaped how these phenomena were differentially experienced in distinctly gendered ways that each led to PTSD/SUD.

**Table 2:** Visualization of theme and subthemes.

Theme	Multiple jeopardy and PTSD/SUD
Shared Thematic Patterns	Violence; silence; coping
Women's Thematic Patterns	Sexual violence; intimate partner violence; racialized gender‐based violence/testimonial injustice; victim‐blaming; institutional minimization of harm/substance use as self‐medication for trauma; constrained access to supportive care
Men's Thematic Patterns	Physical violence; community violence; racialized surveillance and police violence/mental health stigma; norms of masculinity; criminalization of substance use/substance use as emotional suppression and survival strategy; avoidance of formal mental health services

*Note*: Themes represent recurring patterns across participants' accounts regarding how intersecting racialized and gendered oppression shaped trauma, silence, coping, and PTSD/SUD experiences.

Abbreviations: PTSD, posttraumatic stress disorder; SUD, substance use disorder.

### Multiple jeopardy as a structuring condition of PTSD/SUD onset

Our participants' experiences can be viewed through a multiple jeopardy lens, a theory that applies an intersectional framework to suggest that forces of oppression (e.g., racism and sexism) can interact in compounding ways to produce distinct forms of harm for people based on the unique combinations of their identities (King, 1988; Settles & Buchanan, 2014). In this study, racism and sexism were the prevailing forces of oppression that produced PTSD/SUD for people of color in this sample. However, their manifestations differed by gender, such that women's experiences with sexism made them more vulnerable to sexual violence, which participants identified as central to the development of their PTSD. In contrast, men's experiences with sexism made them more vulnerable to gun, gang, or family violence, which contributed to their PTSD. Next, women's combined experiences with racism and sexism served to silence them about their trauma through mechanisms of testimonial injustice and victim blaming, which served to produce their PTSD/SUD. Yet, men's combined experiences with racism and sexism silenced them through norms of masculinity and criminalization, which served to produce their PTSD/SUD. These gender‐differentiated experiences illustrate how the same oppressive forces can operate differently across identity lines and how their effects accumulate over time. Multiple jeopardy makes these layered, identity‐contingent pathways visible.

#### Multiple jeopardy and women's PTSD/SUD: Violence, silence, and coping

Most women endured gender‐based violence (e.g., sexual victimization, intimate/domestic violence, stalking, assault, or a combination), which contributed to the onset of their PTSD. Further, they reported that they did not discuss their experience with others, particularly healthcare professionals, recognizing that women, particularly women of color, are often doubted or blamed for their gender‐based trauma. Therefore, they used substances to cope with their trauma, which eventually led to the onset of their PTSD/SUD.

To illustrate, Maritza, an 18‐year‐old Latina in her first year of college, shared, “I got raped… I couldn't stop thinking about it or dreaming about it, but I didn't feel like I could tell anyone… It's a he said, she said kinda thing, and people *never* believe the girl. I didn't want to go through that… One day, I used some pain pills for a soccer injury… I took them and felt like it [the trauma] didn't matter anymore… I felt better for the first time in a while.” Further, Maritza shared, “It was taking pills or having my life put under a microscope, so I took the pills… Talking about being raped isn't good for a girl's reputation. Everyone is gonna have that question in their mind, like– *did she make it up*?” Maritza's account illustrates how fear of reputational harm and victim‐blaming directed her toward substance use as a means of coping.

Similarly, Audre, a 23‐year‐old multiracial woman racialized as Black shared that she was sexually victimized as a child and as an adult. Starting at a young age, she coped with these assaults with drugs because “people just don't help women with these things… like, no one was gonna believe me [laughs sardonically]. People really liked [the perpetrator] and, I mean, look at the “Me‐Too” movement– these powerful women are coming forward and saying things, and even they're not believed. It's just ingrained early in [a woman's] life– it's a man's world, so like, your body is theirs, and your words don't matter.” Her reflections demonstrate how early awareness of gendered disbelief and patriarchal norms contributed to internalized expectations of silence and shaped substance use as a means of managing trauma in the absence of support.

Further, Bell, a 33‐year‐old multiracial woman from Georgia, racialized as Black, shared, “I was beat up and raped by my ex on numerous occasions… he caused my PTSD for sure…”. Then, without prompting, Bell shared, “But who was I gonna tell? His friends all thought he was a good guy, so they weren't gonna take my side… He made sure that I didn't have any friends… that's how they [abusers] move… anyway, if I told someone, I felt like someone was going to blame me for staying with him for so long in the first place…”. When asked whether Bell believed that the issues she endured were related to her racial or gender identity, she stated “I feel like if I were a man, I wouldn't have ever been raped. It's not even a question that I was raped *because I was a woman*.” Then, Bell stated, “But not just a woman, a *Black woman*… like, Black women are extra vulnerable to rape just because we're some of the lowest [ranked] in society… add that to [the fact that] Black women are [seen as] the problem, not the actual problem [like abuse or violence], you know? It's a known thing that's talked on all over Black Twitter and other spaces… we're blamed for trusting that man or for staying with him or whatever reason people come up with… so it was just a whole lot easier to hit the bottle.” Bell's experience demonstrates that racism and sexism intersected to shape the onset of her PTSD/SUD.

Finally, Alicia, a 24‐year‐old Black woman from New Mexico, shared that one night at a party, she was high with a friend who sexually assaulted her several times. However, Alicia said, “I didn't want to tell anyone… I mean, I just felt like they would blame *me*. I didn't want to tell cops, therapists, or anyone. I felt alone.” When Alicia was asked if she believed her race or gender played a role in her experiences, she emphatically stated, “*Yes!* Black women are expendable. So yeah, in a way, I feel this wouldn't have happened if I were someone else [someone else other than a Black woman].” When questioned about whether she worried that she would be blamed because of her race and gender, she again emphatically stated, “Completely. I know so many other Black women who have gone through what I went through, but they were ignored or were told that they should have avoided the man in the first place…” Alicia's narrative reveals how intersecting systems of racism and sexism constrain access to care and redirect coping toward isolation and substance use rather than support.

Across these accounts, women consistently described how gender‐based violence shaped the onset of their PTSD and how racism‐related victim blaming led them to use substances to cope with their PTSD. While substances offered temporary relief from trauma‐related distress, these structural issues eventually led to SUD. Thus, their narratives reveal how sexism and racism impact women in ways that lead to the onset of PTSD/SUD.

#### Multiple jeopardy and men's PTSD/SUD: Violence, silence, and coping

Most men experienced interpersonal traumas like gang or gun violence, child abuse, or witnessing violence against people they cared about, which led to their PTSD. For example, Martin, a 37‐year‐old Black man from Georgia, shared, “I watched my friends get shot. I was right there next to them. It stuck with me… it *still* sticks to me.” When asked whether matters of race or gender were related to his experience, he shared, “Black men are just thrown into the jungle. Where I grew up, and It's not like I grew up poor or anything, which just goes to… goes to show how *entrenched* this is… where I grew up there were no cops, no like, playgrounds or anything… all we had were… were you're either gonna get involved with the wrong crowd and get shot or do the shooting, *or*… or you're gonna stay inside and hit the books.” He elaborated, “But there was always this thing like the *real man* has the gun in his hand… so I guess I wasn't a real man [laughs]. I hit the books, but my friends, man, my friends didn't.” Martin's experience illustrates how structural racism and rigid masculine norms converge to create environments where trauma is prevalent and normalized.

Similarly, Oscar, a 26‐year‐old Latino from New Mexico, shared, “My mom was illegal, and my dad abused us. He knew she couldn't get help because of her status. Who could I tell because my mom would get in trouble? I felt like I let her down because I couldn't stand up to him, and I couldn't get help either. Watching that growing up really [messed] me up… that's why I have PTSD.” Then, Oscar elaborated on how gender was related: “In Latino culture, the boy is the man of the house. He's the protector. So when I couldn't protect my mom, it added up my mental issues. It's like two things I'm dealing with– seeing that violence [and] dealing with the fact that I was supposed to be able to do something about it [as a man], but couldn't.”

Further, most men in this group shared that they coped with the PTSD brought about by these racialized and gendered forms of violence by using substances, which eventually led to their co‐occurring SUD. To illustrate, Malcom, a 29‐year‐old Black man from Georgia, shared that he endured child abuse and witnessed domestic violence frequently as a child. After leaving home at 18, he knew that his mental health was suffering. “I couldn't form any relationships with people, I was always angry, and I knew my childhood messed me up.” He stated, “… but Black men don't talk about their mental health. It's not a big secret that we don't, so we have to deal with our issues some other way… For me, it was using drugs. I used drugs, and I felt better for a long time. It was the whole culture of silence that caused me to get addicted to drugs.”

In addition, Cesar, a 19‐year‐old Latino from California, shared, “I have a really good family. They're smart… they pushed me to get into college. But I couldn't talk to them about certain things. My dad is old‐school Mexican – he never smiles; he laughs only *sometimes*. I mean, I don't even know what he feels half the time. And that's just normal. I think I'm the same way sometimes. So after it [Cesar's trauma] happened, I couldn't talk to him. And we [Mexican men] don't do therapy… that's how I thought at the time. It was just better to smoke and drink. At least if I got in trouble for that, my dad would probably be like– ‘ at least it wasn't no weird shit.'” When asked if “weird shit” was a reference to getting therapy, Cesar said, “Yeah, my dad still doesn't know. He would think it's weak.”

Yet, stigma around mental health was not the only thing that led men in this sample to use substances to cope with PTSD. The criminalization of men of color's victimization also played a role in promoting their SUD. For instance, Oscar shared, “…because my mom was illegal, who could I tell because mom would get in trouble… plus, I didn't know if I would get arrested if I told someone about my drug use… and it's not like I could find out or anything– no one shares anything in our culture… so you just check out.” Likewise, Martin shared, “It was seeing people get shot that [traumatized] me… I couldn't tell a damn therapist or school counselor or whatever because who are they gonna call [he means the police]? I couldn't trust anyone… so umm… I drank a lot. It helped me… but I used other things too because that's what was available.”

Yet, eventually their substance use escalated into addiction. However, they avoided seeking therapy or formal support, fearing that disclosing their traumas or substance use they would incur legal repercussions. For instance, Deray shared, “… I knew I was addicted, but at that point, was I really willing to risk getting arrested for drugs or ‘cause of street' violence to get help? Nope.” Similarly, Malcom stated, “I couldn't trust anyone with what I had to say, especially because I'm using drugs, man. I didn't know what they [therapists] would report and what they wouldn't…”.

Moreover, men's hesitance to seek help due to criminalization was compounded by concerns of violating masculine norms. Martin stated, “Men don't talk about their feelings. So, going to therapy was a double‐no for me for a long time. I still haven't told my family I'm in therapy because they'd be like, “What do you need therapy for? Man up!” Thus, even as men's substance use escalated into addiction, many avoided therapy or support, fearing that disclosing their struggles would lead to stigma or legal repercussions rather than care. These men's narratives reveal how racialized environments, masculine norms, and criminalization work together to produce PTSD/SUD.

## DISCUSSION

Although violence, silence, and coping appeared in both men's and women's narratives, their experiences varied by gender, making the role of multiple jeopardy in contributing to PTSD/SUD onset visible. Among the women of this sample, sexism functioned to expose them to sexual and gender‐based traumas that caused their PTSD. Their experiences are supported by national data, which shows that sexual trauma is a primary contributor to PTSD in women (Kessler et al., 2014). Further, the women in this sample discussed how racism and sexism interlocked in ways that caused their SUD. They shared the concern that women, and women of color in particular, are often disregarded or blamed when it comes to matters of sexual trauma (Linhares et al., 2023). Therefore, had they come forward about their traumas, they would not have been believed or, in some cases, blamed.^1^ These concerns have been articulated in research as testimonial injustice, an issue whereby a person's credibility is at risk of dismissal due to their identity (Fricker, 2007), and victim blaming, an issue that occurs when a person is held liable for their victimization (Linhares et al., 2023; Ryan, 1976). Research consistently shows that women are highly likely to be discounted or dismissed when they report encounters with victimization (Epstein & Goodman, 2019), and that women of color are more likely than White women to be invalidated or blamed for experiencing assault (Linhares et al., 2023). Consequently, women in this sample coped with their challenges by using substances, which subsequently led them to experience PTSD/SUD.

Next, structural racism played a role in men's trauma by exposing them to gang, gun, or other forms of physical violence, as well as keeping them from seeking help for their traumas due to concerns around criminalization. Their reports correspond with data demonstrating that structural racism via over‐ and under‐policing places men of color at a heightened risk for traumatic experiences like gun violence, and raises their risk of being arrested in relation to those traumatic circumstances (Alexander, 2010; Bailey et al., 2017; Rothstein, 2017). Furthermore, sexism, playing out through rigid masculinity norms that discourage emotional expression and seeking help (Johnson et al., 2024; Williston et al., 2019), led them to use substances to manage distress. However, because substance use is also heavily criminalized among men of color (Cohen et al., 2022; Earp et al., 2021), many expressed feeling trapped in a double‐bind: they could not seek support for their PTSD without compromising their masculinity and risking criminalization, nor could they overcome their SUD without risking being surveilled or policed. The convergence of racialized trauma, gendered stigma, and racist criminalization thus demonstrates how racism and sexism simultaneously influence men of color's PTSD/SUD onset.

Our participants' experiences show that structural and interpersonal racism and sexism are fundamental, interlocking contributors to PTSD/SUD onset. However, despite mounting evidence of racial and gender disparities in PTSD/SUD onset and health outcomes (Emerson et al., 2017), PTSD/SUD research has remained effectively colorblind for the past two and a half decades, with virtually no attention to how race or racism shape symptom onset, expression, or treatment engagement (Reid et al., 2025). Gender‐specific interventions also remain limited and are primarily focused on sexual violence, whereas they fail to address some of the gendered issues identified by participants in this study (Reid et al., 2025).

Moreover, when people of color access care, they frequently encounter microaggressions that retraumatize them, erode trust, and negatively affect symptoms (Hook et al., 2016; Sue & Spanierman, 2020). Further, women of color are often dismissed or pathologized in therapeutic settings (Epstein & Goodman, 2019), and clinicians have shared substance use data with police, enabling arrests (Canning et al., 2021). Indeed, the women and men of color in our sample avoided treatment over these issues: women anticipated being discounted or blamed for their trauma, and men worried that disclosing their substance use or trauma might lead to criminalization. Thus, our findings reveal the importance of continuing to advocate for multicultural competence among clinicians and for research agendas that ensure changes in our systems that allow the needs of people of color to be addressed (Buchanan & Wiklund, 2020).

## IMPLICATIONS FOR COMMUNITY PSYCHOLOGY

A preliminary review of the major US community psychology journals indicates that community psychologists seldom study PTSD/SUD. Community psychology has a strong legacy of focus on addressing social justice issues and their impact on behavioral health (Nelson et al., 2014; Nelson, 2010); therefore, this lack of engagement is unexpected given that PTSD and SUD are closely tied to social injustices. Importantly, community psychologists are well‐equipped to contribute meaningfully to PTSD/SUD research, as their expertise in community‐engaged methodologies can help rectify the field's historical neglect of people of color. PTSD/SUD research has often relied on frameworks that obscure the role of structural oppression, leading to interventions that fail to address the intersecting forces shaping mental health outcomes. Unlike traditional approaches that focus on individual pathology, community psychology foregrounds systemic factors (Gridley & Turner, 2010; Hawe, 2017) and centers the lived experiences of marginalized groups (Campbell et al., 2017; Kidd et al., 2018). By leveraging these strengths, community psychologists can construct interventions that are culturally responsive and attuned to the social conditions that sustain PTSD/SUD disparities. If community psychologists were to engage more significantly with PTSD/SUD, their work could transform interventions and policies to directly address the root causes of PTSD/SUD among oppressed groups.

## LIMITATIONS AND FUTURE DIRECTIONS

While this study provides critical insights into how multiple jeopardy shapes the onset and progression of PTSD/SUD among people of color, several limitations should be acknowledged. Our sample mainly comprised people with a high school education or less, which may have influenced the kinds of issues related to racism and sexism that our participants reported. Indeed, those with higher socioeconomic status may report unique challenges that are equally important to uncover. Therefore, future research should include greater socioeconomic diversity to assess how class interacts with race and gender in shaping PTSD/SUD experiences. Second, our sample size was relatively small (*N* = 23), which allowed for in‐depth exploration of lived experiences but may have limited the breadth of perspectives we captured. A larger sample could reveal additional dimensions of multiple jeopardy that impact PTSD/SUD. Also, future research should explore the experiences of other marginalized groups (e.g., sex and gender minorities, migrants, etc.).

Further, our analysis focused broadly on the intersecting structural and interpersonal effects of racism and sexism. However, participants' narratives may also reflect culturally specific gendered racial scripts discussed in prior literature, such as expectations surrounding emotional stoicism among men of color or the endurance and silencing often imposed upon women of color. Because our sample included multiple racialized groups and was not designed to conduct in‐depth within‐group analyses of culturally specific schemas, these questions warrant further targeted investigation in future research that may more explicitly examine how frameworks such as racialized masculinity norms, machismo, misogyny, or the “strong Black woman” schema intersect with PTSD/SUD trajectories among specific racialized groups.

Additionally, because participants reflected retrospectively on the onset of their PTSD/SUD, their narratives represent meaning‐making processes shaped by memory, recovery status, and current understandings of race, gender, and trauma. Thus, participants' interpretations of how oppression contributed to their PTSD/SUD may have evolved over time and may not fully capture the complexity or temporality of these experiences as they unfolded. Future longitudinal or prospective qualitative research may help clarify how understandings of racism, sexism, trauma, and substance use develop and shift across the life course.

Moreover, although this study identified important shared patterns across participants, grouping diverse racialized communities together under the broader category of “people of color” may obscure important within‐group differences in trauma exposure, coping, stigma, criminalization, and help‐seeking experiences. Future research should examine PTSD/SUD trajectories within specific racialized communities and attend more closely to how histories, migration contexts, cultural norms, and racialization processes shape experiences differently across groups.

Finally, although we randomized people for their follow‐up interviews, we may have had richer details and insights had we not been constrained by funding and been allowed to conduct a follow‐up interview with more or all participants. Future studies should investigate these phenomena with more follow‐up interviews.

## CONCLUSION

Co‐occurring PTSD and SUD research has significantly under‐included people of color and has meaningfully underexamined issues that are known to impact people of color's behavioral health (e.g., racism and sexism). Yet, our study found that these factors were highly relevant to the onset of people of color's PTSD/SUD and that people of color readily noted the link between their experiences and oppression. Moreover, we found that racism and sexism intersected differently across gendered experiences among people of color, shaping distinct pathways into PTSD/SUD. This multiple jeopardy is an important factor for researchers and clinicians to recognize and address if PTSD/SUD research and interventions are to promote health equity. This finding is an opportunity for community psychologists to leverage their values and research approaches to support new health equity approaches to PTSD/SUD research.

## CONFLICT OF INTEREST STATEMENT

The authors declare no conflicts of interest.

## Data Availability

The data are qualitative and contain personal identifying information. Therefore, the data are not publicly available due to privacy or ethical restrictions. The qualitative data generated and analyzed during the current study are not publicly available due to the sensitive nature of participant disclosures and the potential risk of participant identification.

## Footnotes

1 Importantly, the women in this sample did not state an opinion about whether they would be believed or blamed. They were certain that they encounter these experiences. Therefore, we did not hedge their assertions as opinion or perception. Doing so risks subtly undermining participants' authority. Further, reframing their certainty as perception would reproduce the dynamics of dismissal and disbelief they described. We invite readers to confront any reflex to or reinterpret their accounts as opinion, a reflex that mirrors the systemic silencing women often face.
